# Retraction Note: Regardless of inequities in care, terminal anorexia nervosa exists: a response to Sharpe et al.

**DOI:** 10.1186/s40337-023-00839-3

**Published:** 2023-07-17

**Authors:** Joel Yager, Jennifer L. Gaudiani, Jonathan Treem

**Affiliations:** 1 Department of Psychiatry, University of Colorado School of Medicine, Aurora, CO USA; 2 Gaudiani Clinic, Denver, CO USA; 3 Department of General Internal Medicine, University of Colorado School of Medicine, Aurora, CO USA

**Retraction Note : Journal of Eating Disorders (2023) 11:79** 10.1186/s40337-023-00808-w

The authors have retracted this article following discussions with multiple stakeholders. The authors offer their deepest apologies for the distress and the unintended negative impact of this piece.

